# Platelet-targeted thromboprophylaxis with a human serum albumin fusion drug: Preventing thrombosis and reducing cardiac ischemia/reperfusion injurywithout bleeding complications

**DOI:** 10.7150/thno.97517

**Published:** 2024-05-19

**Authors:** Yuyang Song, Laura A. Bienvenu, Viktoria Bongcaron, Shania A. Prijaya, Ana C. Maluenda, Aidan P. G. Walsh, James D. McFayden, Geoffrey A. Pietersz, Karlheinz Peter, Xiaowei Wang

**Affiliations:** 1 Molecular Imaging and Theranostics Laboratory, Baker Heart and Diabetes Institute, Melbourne, VIC, Australia.; 2 Department of Cardiometabolic Health, University of Melbourne, VIC, Australia.; 3 Baker Department of Cardiovascular Research, Translational and Implementation, La Trobe University, Melbourne, Australia.; 4 Atherothrombosis and Vascular Biology Laboratory, Baker Heart and Diabetes Institute, Melbourne, VIC, Australia.; 5 Department of Medicine, Monash University, Melbourne, VIC, Australia.

**Keywords:** anticoagulant, antiplatelet, targeted drug delivery, thromboprophylaxis, thrombosis

## Abstract

**Background:** Myocardial infarction (MI) as a consequence of atherosclerosis-associated acute thrombosis is a leading cause of death and disability globally. Antiplatelet and anticoagulant drugs are standard therapies in preventing and treating MI. However, all clinically used drugs are associated with bleeding complications, which ultimately limits their use in patients with a high risk of bleeding. We have developed a new recombinant drug, targ-HSA-TAP, that combines targeting and specific inhibition of activated platelets as well as anticoagulation. This drug is designed and tested for a prolonged circulating half-life, enabling unique thromboprophylaxis without bleeding complications.

**Methods:** Targ-HSA-TAP combines a single-chain antibody (scFv) that targets activated glycoprotein IIb/IIIa on activated platelets, human serum albumin (HSA) for prolonged circulation, and tick anticoagulant peptide (TAP) for coagulation FX inhibition. A non-binding scFv is employed as a non-targeting control (non-targ-HSA-TAP). Its efficacy was investigated *in vivo* using murine models of acute thrombosis and cardiac ischemia-reperfusion (I/R) injury.

**Results:** Our experiments confirmed the targeting specificity of targ-HSA-TAP to activated platelets and demonstrated effective prevention of platelet aggregation and thrombus formation, as well as FXa inhibition* in vitro*. Thromboprophylactic administration of targ-HSA-TAP subcutaneously in mice prevented occlusion of the carotid artery after ferric chloride injury as compared to non-targ-HSA-TAP and PBS-control treated mice. By comparing the therapeutic outcomes between targ-TAP and targ-HSA-TAP, we demonstrate the significant improvements brought by the HSA fusion in extending the drug's half-life and enhancing its therapeutic window for up to 16 h post-administration. Importantly, tail bleeding time was not prolonged with targ-HSA-TAP in contrast to the clinically used anticoagulant enoxaparin. Furthermore, in a murine model of cardiac I/R injury, mice administered targ-HSA-TAP 10 h before injury demonstrated preserved cardiac function, with significantly higher ejection fraction and fractional shortening, as compared to the non-targ-HSA-TAP and PBS control groups. Advanced strain analysis revealed reduced myocardial deformation and histology confirmed a reduced infarct size in targ-HSA-TAP treated mice compared to control groups.

**Conclusion:** The inclusion of HSA represents a significant advancement in the design of targeted therapeutic agents for thromboprophylaxis. Our activated platelet-targeted targ-HSA-TAP is a highly effective antithrombotic drug with both anticoagulant and antiplatelet effects while retaining normal hemostasis. The long half-life of targ-HSA-TAP provides the unique opportunity to use this antithrombotic drug for more effective, long-lasting and safer anti-thrombotic prophylaxis. In cases where MI occurs, this prophylactic strategy reduces thrombus burden and effectively reduces cardiac I/R injury.

## Introduction

Acute thrombosis is a major cause of death and disability worldwide, which leads to vessel occlusion and resulting ischemic complications such as myocardial infarction (MI). The main disease underlying these events is atherosclerosis, with the rupture of unstable inflamed plaques exposing highly thrombogenic material, leading to platelet activation, aggregation, fibrin formation, and ultimately, clot formation and vessel occlusion 1. While traditional antiplatelet and anticoagulant therapies have been instrumental in preventing and treating thrombosis, these drugs often require high doses to achieve sufficient antithrombotic effects and, especially when used in combination, are directly associated with an increased risk of bleeding 2. For anticoagulant/antithrombotic treatment, oral administration or i.v. injection of factor (F) Xa inhibitors is often used, but it is also linked to bleeding complications 3,4. This potentially fatal side effect limits the wider use of these drugs for thromboprophylaxis, especially in patients with high risk of bleeding, many of whom are also often at high risk of adverse cardiovascular events.

The use of antithrombotic therapies for the secondary prevention of adverse cardiovascular events in patients with cardiovascular disease is predicated on the ability for these therapies to prevent thrombosis in the setting of plaque rupture and thereby prevent subsequent cardiovascular events such as MI. However, cardiovascular events despite antithrombotic therapy are common and occur in up to 18.5% of patients 4. Antithrombotic treatment is known to attenuate infarct size and, even in the case of spontaneous or interventional recanalization, antithrombotic treatment is beneficial. Cardiovascular events such as MI and stroke are often associated with ischemia/reperfusion (I/R) injury, which reflects a thromboinflammatory response triggered by reperfusion to the ischemic organ. The I/R response is typically associated with micro-thrombotic vessel occlusions, resulting in additional irreversible organ damage 5-7. To overcome these issues, we aimed to design an activated platelet-targeted combination drug with antiplatelet and anticoagulant effects and with a long-circulating half-life, allowing for the prevention of thrombosis and, if needed, the treatment of cardiac I/R injury.

Our group has generated and extensively tested single-chain antibodies (scFv) that specifically bind to activated glycoprotein (GP) IIb/IIIa of activated platelets (scFv_Targ_) 8. GPIIb/IIIa is the most abundant receptor on the surface of platelets and it undergoes a conformational change upon platelet activation, switching the conformation from a low- to a high-affinity state for fibrinogen binding 8. We have genetically engineered several scFv-drug constructs and one of the most promising drug candidates that we previously developed is activated platelet-targeted tick anticoagulant peptide (TAP), an innovative factor (F) Xa inhibitor fusion protein 9-11.

We genetically fused scFv_Targ_ to TAP (targ-TAP) to create a novel antiplatelet/anticoagulant drug for the effective treatment of thrombosis 10. We also demonstrated that targ-TAP preserved heart function and reduced infarct size post cardiac I/R injury *in vivo* when injected intravenously (i.v.) at the induction of injury 12. Most importantly, targ-TAP can be given in low systemic doses, preventing bleeding complications, which is highly advantageous as it provides a novel approach for thromboprophylaxis 10,12. However, we recognized that targ-TAP has a low molecular weight and is rapidly cleared from the circulation after i.v. and subcutaneous (s.c.) administration 10. This fast clearance limits its clinical translatability as a thromboprophylactic drug.

Given the previously established therapeutic efficacy of targ-TAP, our research therefore focuses on enhancing its clinical applicability by prolonging its circulation half-life, which is critical for achieving sustained therapeutic and in particular prophylactic efficacy. We aimed to increase the half-life by including human serum albumin (HSA) in this scFv drug conjugate to allow its use as a prophylactic drug. HSA is the most abundant plasma protein in humans, acts as a natural transport protein, and has a long-circulating half-life; these unique properties make it ideal to be employed for drug modification, thereby improving pharmacokinetics and pharmacodynamics 13-15. By genetically fusing HSA to targ-TAP, we exploit the intrinsic properties of HSA to stabilize the antibody conjugate by preventing premature degradation and rapid clearance from the bloodstream. Its targeted mechanism of action reduces systemic side effects, enhancing its safety profile, making it a considerably safer option for long-term thromboprophylaxis compared to currently clinically used antithrombotic drugs, particularly in patients at high risk for thrombotic and bleeding events.

In this study, we characterize the antiplatelet and anticoagulant activity of targ-HSA-TAP and provide *in vivo* proof of concept for the benefits of thromboprophylaxis in preventing acute thrombosis and reducing I/R injury post-MI without bleeding complications in murine models. We demonstrate that the benefits of our long-circulating targ-HSA-TAP extend beyond mere clot prevention. In instances where thrombosis develops despite thromboprophylaxis, resulting in MI, targ-HSA-TAP can provide an additional therapeutic dimension by mitigating I/R injury, thereby potentially reducing the extent of myocardial damage. By providing comprehensive management of both clot formation and tissue damage, targ-HSA-TAP represents a significant advancement in the field of cardiovascular thromboprophylaxis, with the potential to improve patient outcomes and redefine treatment paradigms.

## Methods

### Cloning, expression, and purification

The DNA sequences for targ-HSA-TAP and non-targ-HSA-TAP were designed for expression using the pMT vector and purchased (GeneArt gene synthesis; Thermo Fisher Scientific, USA). The antibody drug constructs were expressed in Schneider 2 *Drosophila* cells via dimethyldioctadecylammonium bromide (DDAB) and copper sulfate induction occurred on Day 4 10. Three days later, the cells were harvested and centrifuged. The supernatant was filtered and purified using a copper extraction column (GE HealthCare Life Science, USA). Proteins were collected, dialyzed, and then purified via fast protein liquid chromatography using the AKTA Pure system (GE HealthCare Life Sciences, USA) and a nickel-based metal affinity column (Qiagen, The Netherlands) where proteins containing a His-tag were captured.

### SDS-PAGE and western blot

The purity of the antibody drug constructs (10 μg/well) was determined via SDS-PAGE and an anti-His-tag antibody horseradish peroxidase (Roche, Switzerland) was used to detect the proteins using the Syngene G:BOX Gel Documentation System (Synoptics, USA).

### *In vitro* platelet binding assays via flow cytometry

Human blood was collected from healthy volunteers as per ethics approval from the Alfred Human Ethics Committee (Project No: 627/17). Platelet-rich plasma (PRP) was separated from human blood by centrifugation and platelets were activated with 20 μM adenosine diphosphate (ADP). Platelet activation and binding of activated GPIIb/IIIa receptors were confirmed using PAC-1 FITC and CD-62P PE (BD Biosciences, USA) staining 11. For direct binding assays, penta-His Alexa Fluor 488 (Qiagen, The Netherlands) was used as a secondary antibody for targ-TAP, targ-HSA-TAP, or non-targ-TAP. A competitive binding assay between PAC-1 and targ-HSA-TAP was performed to prove their binding to activated GPIIb/IIIa 11. Flow cytometry was performed on a BD FACS Canto II flow cytometer (BD Biosciences, USA) and the mean fluorescence intensity (MFI) was determined for quantitative measurements.

### Platelet aggregation

96-well light transmission aggregometry using human PRP was performed as previously reported 10. Briefly, 100 μl of PRP was incubated with targ-TAP, targ-HSA-TAP, or non-targ-TAP prior to being activated with 10 μM ADP. Readout measurements were obtained over 30 min.

### Anti-FXa assay

The inhibition of FXa by the purified proteins was determined using the chromogenic substrate Spectrozyme FXa (Sekisui Diagnostics, USA), as per the manufacturer's instructions. Briefly, 10 μM of antibody drug construct and 250 nM human FXa were incubated at 37 °C for 30 min, after which Spectrozyme FXa was added. Reactions were stopped using acetic acid, and their absorbance was measured at 405 nm using the Benchmark Plus microplate spectrophotometer (Bio-Rad Laboratories, USA). Absorbance values generated in the vehicle control were considered to have minimum FXa activity and results are presented as percentage inhibition of this activity.

### Animal experiments

All animals were sourced from Alfred Medical Research and Education Precinct Animal Services and maintained as per the animal ethical guidelines at the Baker Heart and Diabetes Institute, Melbourne, Australia, according to the Alfred Plus Alliance Animal Ethics Committee (No. E/1950/2019/B). Animals were randomized to receive controls or scFv drug fusion constructs, including targ-HSA-TAP, non-targ-HSA-TAP, targ-TAP, non-targ-TAP, PBS control, and enoxaparin control. All animal procedures were performed in accordance with ethical and institutional guidelines at the Baker Heart and Diabetes Institute, Melbourne, Australia.

### Acute thrombosis model of carotid artery induced by FeCl_3_

C57BL/6 mice (20-25 g) were randomized to receive controls or experimental drugs, administered at three different timepoints: 1) i.v. at 5 min; or 2) s.c. at 4 h; or 3) s.c. at 16 h, before acute thrombosis induction. Mice were injected intraperitoneally with a combination of ketamine (100 mg/kg) and xylazine (5 mg/kg) before a small incision was made to expose the left common carotid artery. Thrombus formation was induced on the left carotid artery using a filter paper saturated with 5% FeCl_3_ for 3 min. For quantitative measurements, a T106 flowmeter (Transonic System, USA) and nano Doppler-flow probe (MA0.5VB FLEX, Transonic System, USA) were used to record the blood flow for 30 min; occlusion was defined as less than 0.2 mL/min for over 30 s.

### Myocardial infarction I/R model

C57BL/6 mice (20-25 g) were randomized to receive controls or experimental drugs, administered s.c. 10 h before surgery. Mice were injected intraperitoneally with a combination of ketamine (100 mg/kg), xylazine (5 mg/kg), and atropine (1 mg/kg). After endotracheal intubation, the left anterior descending (LAD) artery was ligated for 1 h before reperfusion 6,12. After reperfusion, atipamezole (0.2 mg/kg) was injected s.c. to help the mice to recover from anesthesia.

### Echocardiography

Echocardiography was performed at baseline before injury and at Week 4 following I/R injury, using the Vevo 2100 system and 22-55 MHz MS 550D transducer (FUJIFILM VisualSonics Inc., Canada) 6,12. Parasternal long-axis and parasternal short-axis views were recorded.

### Histology

Mice were culled at Week 4 following cardiac I/R injury. Briefly, post-anesthesia 3% Evans blue dye was injected into the circulation after ligation of the LAD. The heart was collected for histological analysis. Further staining with 1% triphenyltetrazolium chloride (TTC) was performed before imaging analysis using the Image-Pro Analyzer 7.0.

### Tail bleeding time

C57BL/6 mice (20-25 g) were randomized to receive controls or experimental drugs, administered at two different timepoints: 1) i.v. at 5 min; or 2) s.c. at 4 h. Mice were anesthetized intraperitoneally with ketamine (100 mg/kg) and xylazine (5 mg/kg) before tail transection. Bleeding was monitored without contacting the wound and time was recorded when flow ceased for 30 s.

### Statistical analysis

Sample sizes for animal studies were calculated using SigmaStat, based on results from our previous study 6,10,12. All quantitative data are reported as mean ± standard deviation (SD). Evaluation of outliers was performed using Grubbs' method. All data were assessed for normality using the Shapiro-Wilk test. The F-test was utilized to assess equal variance for data with two groups, while the Brown-Forsythe test was employed for data with more than two groups. Statistical analysis for two groups with normality and equal variance was conducted using Student's t-test, whereas for those with normality but not equal variance, Welch's t-test was applied. One-way ANOVA were used where there was more than two groups with one independent variable and parametric data, followed by post-hoc analysis using Tukey's test. In cases where normality was not met, Kruskal-Willis test with Dunn's multiple comparisons was employed. For situations with more than two groups and one independent variable but without equal variance, the Brown-Forsythe with Dunnett's T3 multiple comparisons were applied. Two-way ANOVA was used where there were more than two groups with two independent variables and parametric data, followed by Sidak's post-hoc test. Sample size (n) is included in the figure legends for each statistical analysis. A P-value of < 0.05 was considered significant. Statistical analysis was performed using Prism 9 (version 9.1.0; GraphPad Software, USA).

## Results

### Production of targ-HSA-TAP and non-targ-HSA-TAP

**Figure 1:**
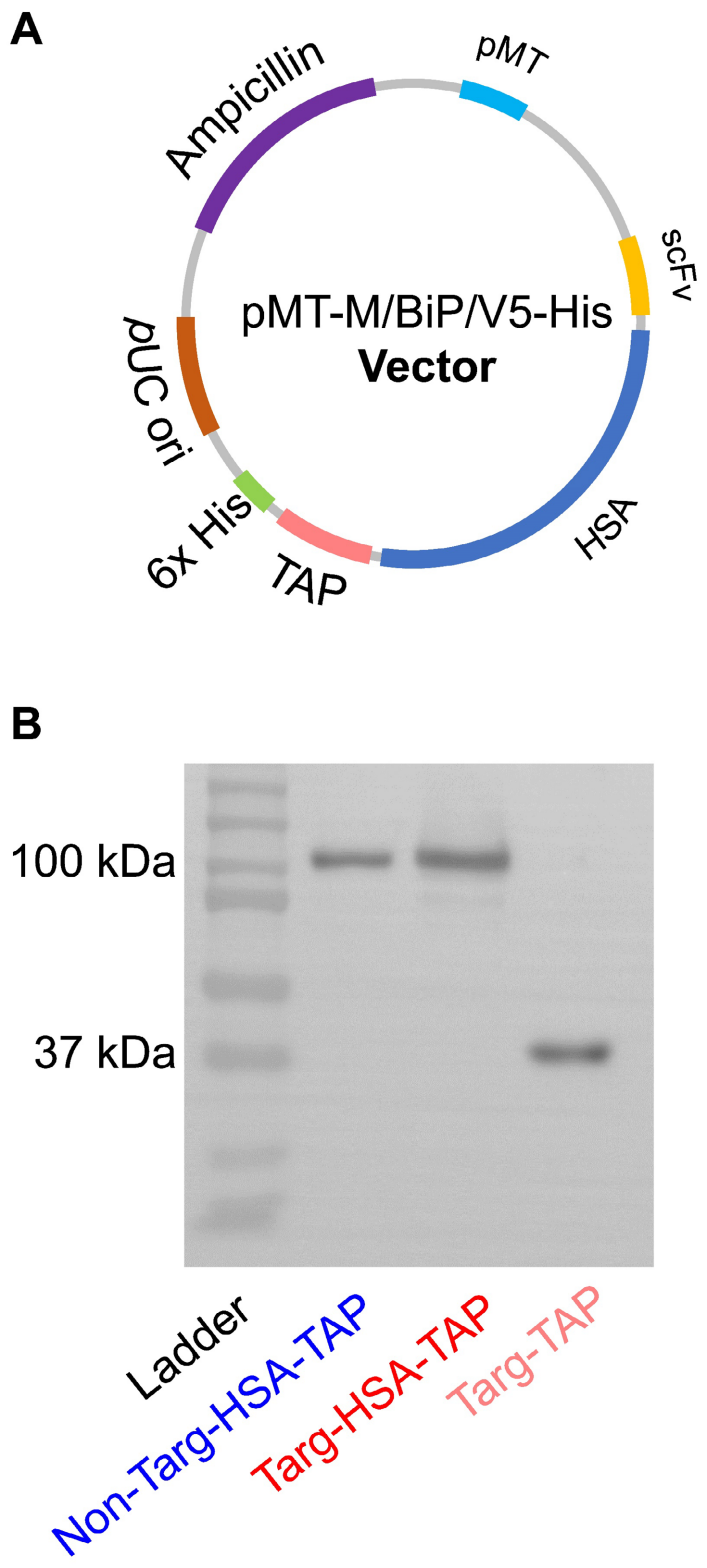
** Expression and purification of targ-HSA-TAP and non-targ-HSA-TAP. A.** Plasmid map of targ-HSA-TAP constructs in pMT. **B.** Western blot (anti-His-HRP) analysis of non-targ-HSA-TAP (100 kDa), targ-HSA-TAP (100 kDa), and targ-TAP (37 kDa).

Generation of a dual-targeted antiplatelet/anticoagulant with an extended circulatory half-life is based on the inclusion of HSA between an activated GPIIb/IIIa-targeting single-chain component (scFv_Targ_) and TAP, a potent factor Xa inhibitor (targ-HSA-TAP). With the incorporation of HSA, we aimed to prolong circulation time, allowing the GPIIb/IIIa-targeting scFv_Targ_ to make the construct home in on activated platelets and increase the localized potency of TAP, thereby providing a longer lasting prophylactic effect. A non-binding scFv-drug fusion protein was also constructed as a control (non-targ-HSA-TAP). The constructs were generated in pMT/BiP/V5-His plasmid (Figure 1A). After production in *Drosophila* S2 cells, the proteins were purified through a copper extraction column, followed by nickel-based affinity chromatography 10. Western blot was performed with anti-His-HRP antibody to confirm the successful production of both targ-HSA-TAP and non-targ-HSA-TAP at 100 kDa, as well as non-modified targ-TAP at 37 kDa (Figure 1B).

### Targ-HSA-TAP binds and blocks activated GPIIb/IIIa receptors

**Figure 2:**
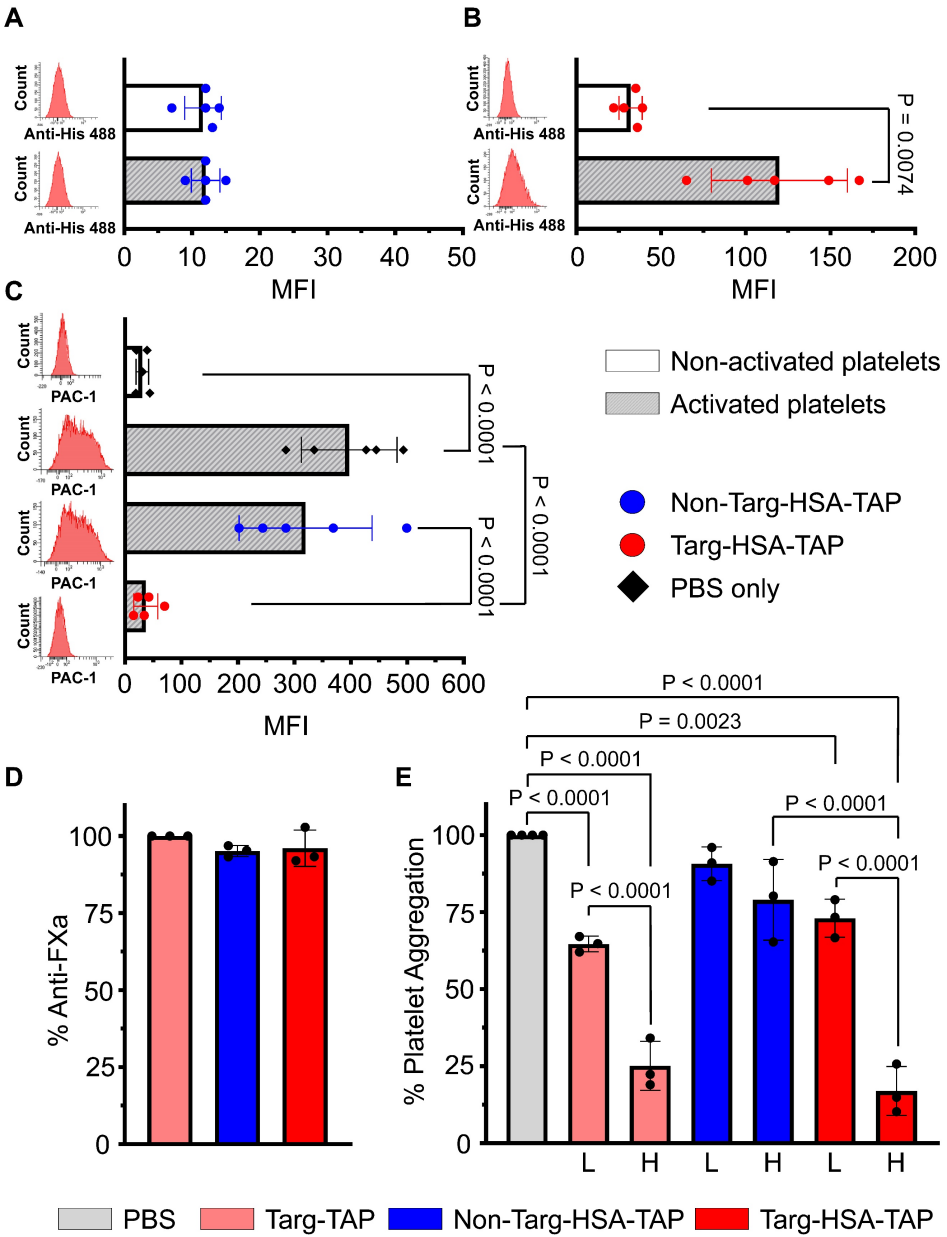
** Targ-HSA-TAP demonstrated antiplatelet and anticoagulant effects.** Flow cytometry assay was conducted using anti-Penta-His AlexaFluor 488 antibody, which binds our constructs (A-C). Platelets were either non-activated or activated with ADP. Bar charts display mean fluorescence intensity (MFI) values of 5 independent experiments. Representative histograms in red are shown on the left of each graph. **A.** Non-targ-HSA-TAP (0.2 μg/mL) resulted in no binding and no shift in histograms, while **B.** Targ-HSA-TAP (0.2 μg/mL) bound to activated platelets but not to non-activated platelets. **C.** Competitive assays using PAC1-FITC bound to activated platelets when incubated with PBS (without constructs) or non-targ-HSA-TAP (5 μg/mL). However, PAC1-FITC failed to bind to activated platelets in the presence of targ-HSA-TAP (5 μg/mL). **D.** Bar chart shows percentage inhibition of FXa after addition of targ-TAP, non-targ-HSA-TAP, and targ-HSA-TAP. Both non-targ-HSA-TAP and targ-HSA-TAP exhibited equivalent anti-FXa activity as the equimolar amount of targ-TAP (n = 3). **E.** 96-well plate light transmission aggregometry demonstrated the antiplatelet effect of targ-HSA-TAP. Bar chart shows percentage aggregometry after addition of ADP. A high concentration of targ-TAP (15 μg/mL) and the equimolar amount of targ-HSA-TAP (39.75 μg/mL) demonstrated strong inhibition of platelet aggregation as opposed to non-targ-HSA-TAP (39.75 μg/mL), which resulted in no inhibition (n = 3). Similarly, a lower concentration of targ-TAP (5 μg/mL) and the equimolar amount of targ-HSA-TAP (13.25 μg/mL) significantly reduced platelet aggregation as compared to the PBS group, while non-targ-HSA-TAP did not. Numerical results shown as mean ± SD. Data in A analyzed using Student's t-test. Data in B analyzed using Welch's t-test. Data in C-E analyzed using one-way ANOVA with Tukey's post-test analysis. L, low concentration; H, high concentration.

The binding of targ-HSA-TAP to activated platelets was demonstrated via flow cytometry. Platelet activation using 10 μM ADP was confirmed by the activation-specific binding of PAC1-FITC, which resulted in a rightward shift on the histogram and a significant increase in MFI as compared to non-activated resting platelets (Figure S1A; 615.4 ± 370.0 *vs.* 20.0 ± 7.1; P = 0.0228). Similar results were obtained using CD62P-PE, another platelet activation-specific marker (Figure S1B; 264.4 ± 187.1 *vs.* 23.6 ± 16.0; P = 0.0003). Using an anti-His Alexa 488, we detected no difference in MFI when non-targ-HSA-TAP was incubated with platelets (Figure 2A; 11.6 ± 2.7 non-activated *vs.* 12.0 ± 2.1 activated; P = ns). In contrast, our targeted fusion construct, targ-HSA-TAP, at a concentration of 0.2 μg/mL, showed a significant increase in MFI when added to activated platelets (Figure 2B; 119.8 ± 40.1 *vs.* 32.0 ± 6.9 non-activated platelets; P = 0.0074). Similar results were demonstrated using higher concentrations of our constructs (0.5 μg/mL to 5 μg/mL; Figure S2). Overall, we demonstrated that the inclusion of HSA does not affect the targeting capability of scFv_Targ_ to activated platelets.

To demonstrate that targ-HSA-TAP binds competitively to the PAC1-FITC binding site on activated GPIIb/IIIa receptors on activated platelets, we first added our constructs to activated platelets to saturate the GPIIb/IIIa binding sites before adding PAC1-FITC. Preincubation with non-targ-HSA-TAP (5 μg/mL) did not affect the binding of PAC1-FITC to activated platelets (Figure 2C; 319.8 ± 117.7 *vs.* 397.0 ± 84.9; P = ns), whereas preincubation with targ-HSA-TAP (5 μg/mL) prevented PAC1-FITC binding (Figure 2C; 36.8 ± 21.2 *vs.* 397.0 ± 84.9; P < 0.0001). Concentration-dependent competitive binding of targ-HSA-TAP was observed using a lower dose of the targeted drug (2 μg/mL), where the inability to saturate all activated GPIIb/IIIa receptors allowed for less PAC1-FITC binding (Figure S3). Therefore, we demonstrated that targ-HSA-TAP and PAC1-FITC compete for activated GPIIb/IIIa receptors on activated platelets.

### Both targ-HSA-TAP and non-targ-HSA-TAP exhibit anti-FXa activity

We characterized the anti-FXa potency of the TAP component of the platelet-targeted drugs. Using a chromogenic Xa-specific substrate, we demonstrated that the activity of our newly generated constructs, targ-HSA-TAP and non-targ-HSA-TAP, exhibited equivalent FXa inhibition as the parent protein, targ-TAP 10. All three drugs (10 μM) have similar potency (Figure 2D; 96.0 ± 5.9 *vs.* 95.1 ± 1.8 *vs.* 100.0 ± 0.0, respectively; P = ns).

### In vitro evaluation of targ-HSA-TAP effect on platelet aggregation

Light transmission aggregometry was performed to examine the capability of our targeted drugs to inhibit platelet aggregation after ADP stimulation. At a high concentration, targ-TAP (15 µg/mL) and the equimolar dose of targ-HSA-TAP (39.75 µg/mL) demonstrated strong inhibition of platelet aggregation as compared to the non-targ-HSA-TAP and PBS control (Figure 2E; 25.1 ± 7.9 *vs.* 16.9 ± 7.9 *vs.* 79.0 ± 13.1 *vs.* 100.0 ± 0.0 respectively; P < 0.0001). Similar results were obtained for lower doses (Figure 2E; targ-TAP at 5 µg/mL and targ-HSA-TAP at 13.25 µg/mL). No difference in aggregation was measured for both high and low doses of non-targ-HSA-TAP when compared to the PBS control (Figure 2E; 79.0 ± 13.1 *vs.* 90.7 ± 5.5 *vs.* 100.0 ± 0.0; P = ns).

### Targ-HSA-TAP provides longer lasting therapeutic benefits for arterial thrombosis in mice compared to targ-TAP

A ferric chloride-induced (FeCl_3_-induced) arterial thrombosis model of the left common carotid artery was used to investigate the therapeutic and prophylactic effects of targ-HSA-TAP 11. A nano-Doppler flow probe was placed under the carotid artery to measure blood flow and the area under the curve (AUC) was calculated for quantitative analysis. More flow resulted in higher AUC. Due to the difference in molecular weight (targ-HSA-TAP at 100 kDa and targ-TAP at 37 kDa), equimolar doses were calculated for this experiment.

**Figure 3:**
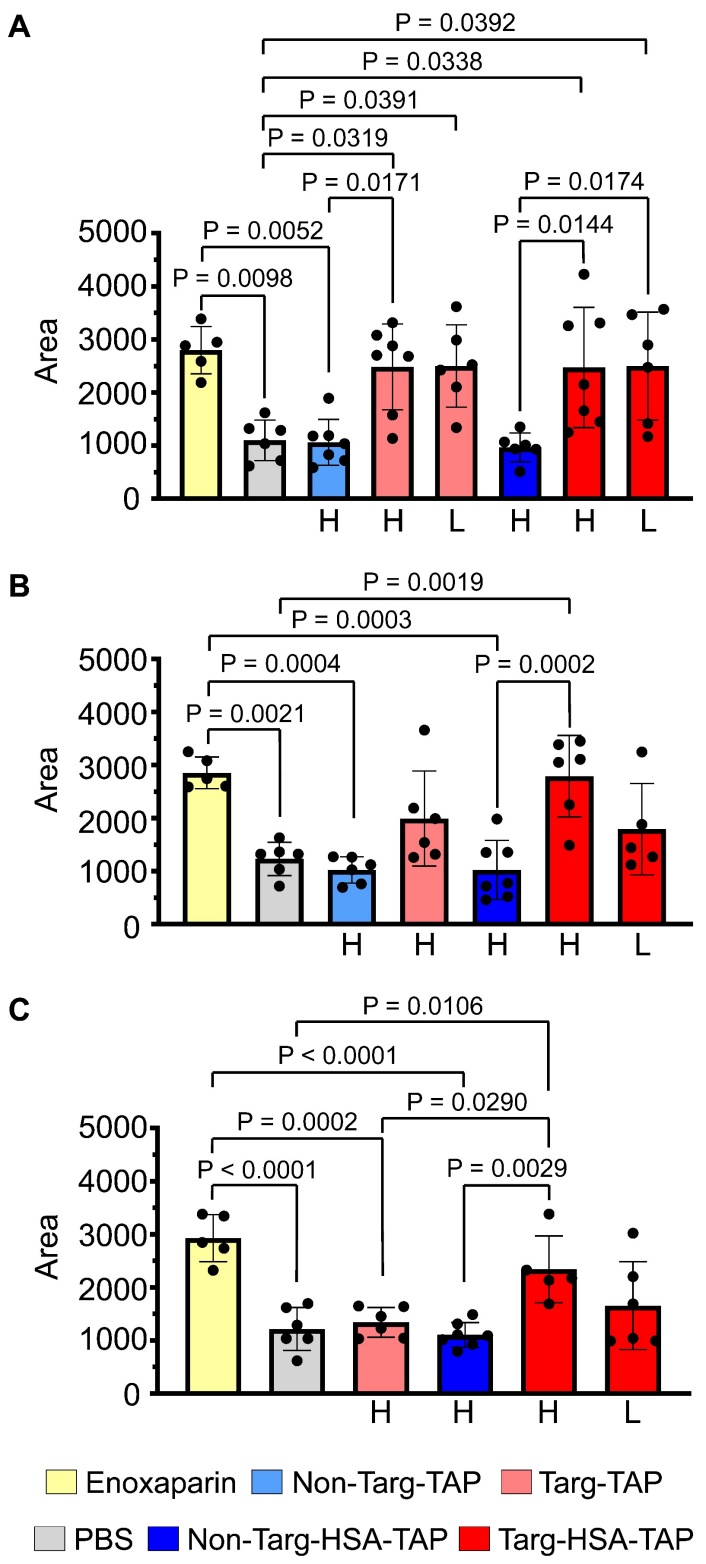
** Targ-HSA-TAP targeted arterial thrombus and inhibited occlusion in mice to 16 h.** Experimental drugs and PBS were administered before inducing thrombosis by 10% ferric chloride at the carotid artery. Two different doses were used for targ-TAP (low dose of 0.03 μg/g BW and high dose of 0.3 μg/g BW); therefore the equimolar amount of targ-HSA-TAP equates to 0.08 μg/g BW and 0.8 μg/g BW, respectively. We only included a high dose for non-targ-TAP at 0.3 μg/g BW and an equimolar amount of non-targ-HSA-TAP at 0.8 μg/g BW. A nano-Doppler flow probe was used to record the blood flow. Area under curve (AUC) was calculated to quantify degree of vessel reopening. **A.** Targ-TAP and targ-HSA-TAP at low and high doses increased AUC when injected i.v. 5 min before injury, n = 5-7. Antithrombotic effects of both targ-TAP and targ-HSA-TAP were comparable to clinically used enoxaparin at 10 μg/g BW. No effect was measured for non-targeted constructs (non-targ-TAP and non-targ-HSA-TAP). **B.** S.c. administration of enoxaparin and a high dose of targ-HSA-TAP 4 h prior to induction of thrombi resulted in similar thromboprophalytic effects, n = 5-7. A high dose of targ-TAP and a low dose of targ-HSA-TAP resulted in trends of increased AUC but were not significant compared to PBS control and enoxaparin. **C.** Longer term prophylaxis properties of targ-HSA-TAP were investigated by injecting it into mice s.c. 16 h before thrombus induction, resulting in increased AUC as compared to PBS and non-targ-HSA-TAP groups, n = 5-7. Numerical results shown as mean ± SD. Data in A-C analyzed using one-way ANOVA with Tukey's post-test analysis. L, low dose; H, high dose.

When the drugs were injected i.v. 5 min before injury, the mice treated with clinically used low-molecular-weight heparin, enoxaparin (10 μg/g), showed a significant increase in AUC as compared to the PBS control (Figure 3A; 2798 ± 445.1 *vs.* 1100 ± 383.8; P = 0.0098). Using the parent proteins in this experiment, we demonstrated that a high dose of non-targ-TAP (0.3 μg/g body weight [BW]) and the equimolar dose of non-targ-HSA-TAP (0.8 μg/g BW) did not prevent thrombosis and resulted in similar AUC to that of the PBS control (Figure 3A; 1061 ± 431.8 *vs.* 968 ± 271.5 *vs.* 1100 ± 383.8 respectively; P = ns). In contrast, significant increases in AUC were measured in both groups of targ-TAP using both a high dose (0.3 μg/g BW) and a low dose (0.03 μg/g BW), as compared to the PBS control (Figure 3A; 2481 ± 808.6 *vs.* 2499 ± 774.3 *vs.* 1100 ± 383.8; P = 0.0319 and P = 0.0391 respectively). Targ-HSA-TAP, at a high dose of 0.8 μg/g BW and a low dose of 0.08 μg/g BW, resulted in significant increases in AUC in a similar manner when compared to its non-targ-HSA-TAP counterpart (Figure 3A; 2472 ± 1133 *vs.* 2498 ± 1018 *vs.* 968 ± 271.5; P = 0.0144 and P = 0.0174 respectively). No difference was observed between different concentrations of targ-TAP and targ-HSA-TAP as compared to the enoxaparin-treated group (P = ns).

We then assessed the short-term prophylactic properties of our drugs by s.c. injection 4 h prior to injury. Enoxaparin maintained its antithrombotic effect and resulted in a significant increase in AUC as compared to the PBS control (Figure 3B; 2852 ± 298.6 *vs.* 1232 ± 313.8; P = 0.0021). High doses of non-targ-TAP and non-targ-HSA-TAP demonstrated no antithrombotic effects when compared to the PBS control (Figure 3B; 1023 ± 247.3 *vs.* 1023 ± 557.4 *vs.* 1232 ± 313.8 respectively; P = ns). The high dose of targ-TAP and the low dose of targ-HSA-TAP both had increased trends but not significant increments in AUC when compared to the PBS control (Figure 3B; 1991 ± 895.2 *vs.* 1792 ± 862.1 *vs.* 1232 ± 313.8 respectively; P = ns). Mice treated with high-dose targ-HSA-TAP maintained significantly larger AUC as compared to the non-targ-HSA-TAP and PBS controls, demonstrating its thromboprophylactic property at 4 h (Figure 3B; 2789 ± 767.4 *vs.* 1023 ± 557.4 *vs.* 1232 ± 313.8; P = 0.0002 and P = 0.0019 respectively).

Next, we investigated the potential of our novel drug candidate to provide longer term prophylaxis by injecting the mice s.c. 16 h before thrombus induction. As expected, both the high dose of targ-TAP and the low dose of targ-HSA-TAP did not increase AUC as compared to the PBS control (Figure 3C; 1340 ± 280.8 *vs.* 1655 ± 827.6 *vs.* 1215 ± 405.0; P = ns). In contrast, the high dose of targ-HSA-TAP resulted in a significantly higher AUC as compared to the non-targ-HSA-TAP and PBS groups (Figure 3C; 2340 ± 628.4 *vs.* 1104 ± 233.2 *vs.* 1215 ± 405.0; P = 0.0029 and P = 0.0106 respectively). Importantly, targ-HSA-TAP was as potent as enoxaparin (Figure 3C; 2340 ± 628.4 *vs.* 2925 ± 443.7; P = ns), demonstrating its usefulness for thromboprophylaxis in an acute *in vivo* thrombosis model.

### Targ-HSA-TAP does not increase bleeding risk

**Figure 4:**
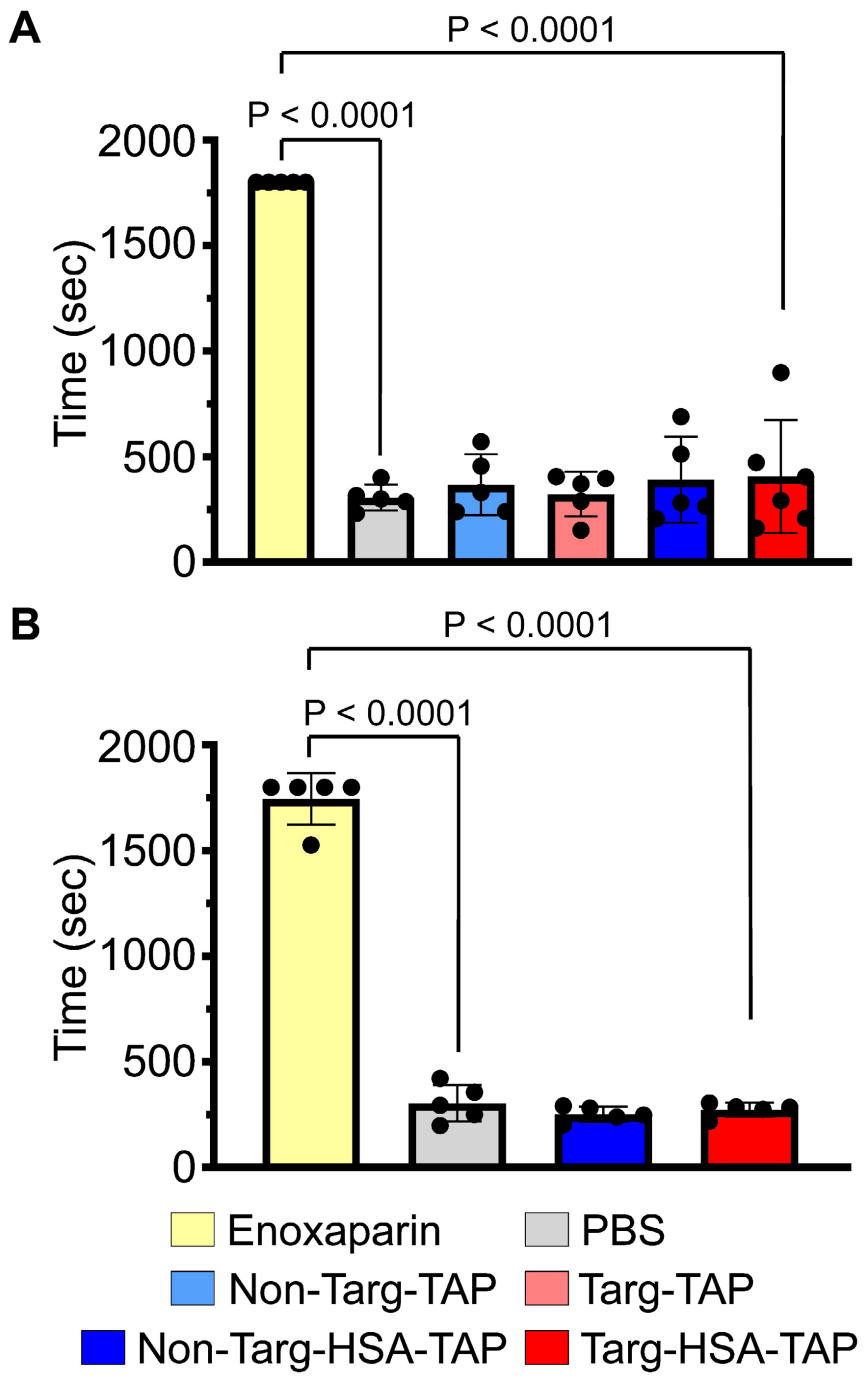
** Bleeding time in mice determined by tail transection demonstrated that targ-HSA-TAP did not prolong bleeding time at the effective dose (0.8 μg/g BW). A.** Clinically used enoxaparin at 10 μg/g BW demonstrated significantly prolonged bleeding time as compared with PBS controls when injected i.v. 5 min before tail transection, n = 5. Non-targ-TAP and targ-TAP (both at 0.3 μg/g BW) and equimolar amounts of non-targ-HSA-TAP and targ-HSA-TAP (both at 0.8 μg/g BW) did not increase bleeding time. **B.** S.c. injection of clinically used enoxaparin at 10 μg/g BW 4 h prior to tail transection significantly prolonged bleeding time in mice as compared to PBS controls, whereas both non-targ-HSA-TAP and targ-HSA-TAP did not result in bleeding, n = 5. Numerical results shown as mean ± SD. Assays analyzed using one-way ANOVA with Tukey's post-test analysis.

Current clinically used antithrombotic drugs are associated with bleeding side effects. We confirmed that the injection of enoxaparin i.v. 5 min prior to tail transection resulted in a significantly increased bleeding time (Figure 4A; 1800 ± 0.0 *vs.* 306 ± 61.1 PBS control, 367 ± 144.1; P < 0.0001). Comparing all other treatment groups, non-targ-TAP, targ-TAP, non-targ-HSA-TAP and targ-HSA-TAP, we found no increases in bleeding time when compared to the PBS control (Figure 4A; 367 ± 144.1 non-targ-TAP, 322 ± 106.4 targ-TAP, 390 ± 203.7 non-targ-HSA-TAP, 406 ± 267.8 targ-HSA-TAP *vs.* 306 ± 61.1 PBS; P < 0.0001). Similar results were obtained when drugs were injected s.c. 4 h before tail transection, when a significantly prolonged bleeding time was induced by enoxaparin compared to the PBS control and our constructs (Figure 4B; 1745 ± 122.5 enoxaparin *vs.* 303 ± 87.1 PBS, 251 ± 36.0 non-targ-HSA-TAP, 273 ± 32.7 targ-HSA-TAP; P < 0.0001). These results indicate that our novel fusion constructs do not increase bleeding risk and, therefore, targ-HSA-TAP can be used for a thromboprophylaxis strategy effectively and safely.

### Targ-HSA-TAP prevents cardiac I/R injury and preserves heart function post-MI

**Figure 5:**
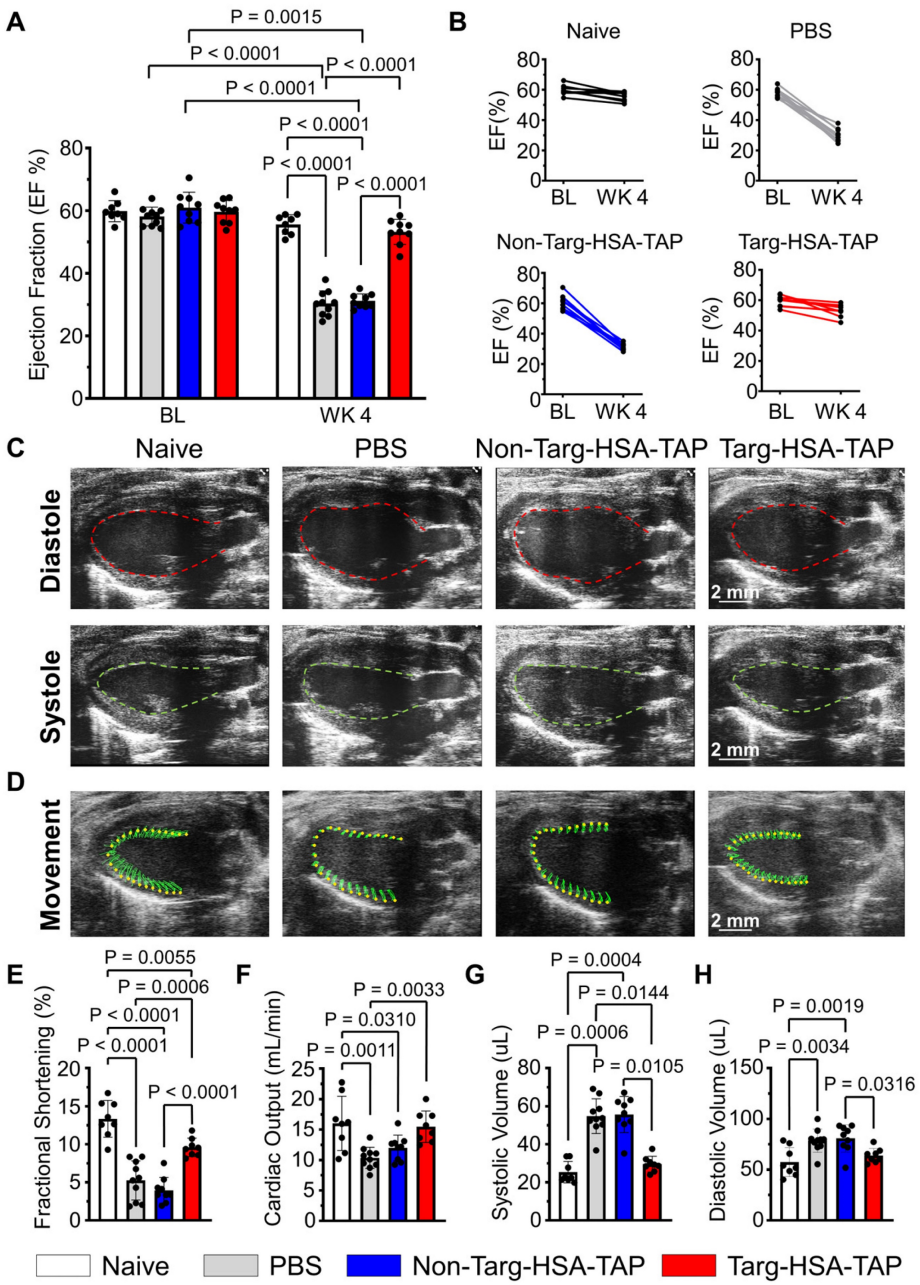
** Targ-HSA-TAP treatment preserved myocardial function in a mouse model of ischemia/reperfusion (I/R).** Cardiac parameters were measured from parasternal long-axis B-mode images at baseline (BL) and 4 weeks post-I/R (WK 4). **A.** Ejection fraction (EF) at baseline was similar between all 4 groups, n = 8-10. Week 4 showed significant decreases in EF for PBS and non-targ-HSA-TAP groups, but targ-HSA-TAP treated animals preserved their EF. **B.** EF for individual animals of each treatment group at baseline and 4 weeks post-I/R. **C.** Representative ultrasound images showing hearts at diastole and systole. **D.** Representative ultrasound images showing movement of cardiac walls. Other cardiac parameters measured include: **E.** Fractional shortening (FS); **F.** Cardiac output (CO);** G.** Systolic volume; and **H.** Diastolic volume, n = 8-10. Numerical results shown as mean ± SD. Data in A analyzed using repeated measures two-way ANOVA followed by Sidak's multiple comparison test. Data in E, F, and H analyzed using one-way ANOVA with Tukey's post-test analysis. Data in G analyzed using Kruskal-Wallis test with Dunn's multiple comparisons.

In a second approach, we investigated whether targ-HSA-TAP can be used prophylactically to preserve cardiac function in a myocardial I/R injury murine model, so the drugs were injected s.c. 10 h prior to injury. Prior to the experiments, baseline echocardiography was performed (Figure S4) and we confirmed that the ejection fractions (EF) were similar for all mice (Figure 5A; 59.9 ± 3.4 naïve *vs.* 58.1 ± 3.0 PBS *vs.* 61.0 ± 4.9 non-targ-HSA-TAP *vs.* 59.7 ± 3.6 targ-HSA-TAP respectively; P = ns). Measurement of EF 4 weeks post-I/R showed significant decreases for mice treated with PBS or non-targ-HSA-TAP as compared to the naïve group (Figure 5A; 30.4 ± 4.0* vs.* 31.2 ± 2.3* vs.* 55.6 ± 3.1 respectively; P < 0.0001). Most importantly, targ-HSA-TAP treated mice had preserved EF and this was not different from the naïve group (Figure 5A; 53.2 ± 4.0 *vs.* 55.6 ± 3.1; P = ns). Comparing the EF at baseline to Week 4, we proved that there was no difference for the naïve group (Figure 5A; 59.9 ± 3.4 *vs.* 55.6 ± 3.1; P = ns). There was a small decrease in EF for the group treated with targ-HSA-TAP (Figure 5A; 59.7 ± 3.6 *vs.* 53.2 ± 4.0; P = 0.0015), which can be attributed to the fact that the mice underwent myocardial I/R injury, Notably, the EF was preserved to a level similar to that of the naïve group. Significant decreases were measured for the PBS group (Figure 5A; 58.1 ± 3.0 *vs.* 30.4 ± 4.0; P < 0.0001) and the non-targ-HSA-TAP group (Figure 5A; 61.0 ± 4.9 *vs.* 31.2 ± 2.3; P < 0.0001). Figure 5B shows the EF of individual animals at baseline and at Week 4 for all experimental groups. Figure 5C provides representative ultrasound images at diasytole and systole for each group and Figure 5D provides representative ultrasound images showing wall movement (Video S1-4).

Fractional shortening (FS), which represents the heart's muscular contractility, was also measured. At 4 weeks post-I/R, FS results correlated with EF measurements; targ-HSA-TAP treatment preserved FS, whereas significant reductions were observed in the PBS and non-targ-HSA-TAP groups (Figure 5E; 9.6 ± 1.2 *vs.* 5.2 ± 2.6 *vs.* 4.0 ± 1.7; P = 0.0006 and P < 0.0001 respectively). Cardiac output (CO) was not significantly different between the targ-HSA-TAP and naïve groups 4 weeks after I/R injury (Figure 5F; 15.5 ± 2.6 *vs.* 16.0 ± 4.5; P = ns). Mice given PBS and non-targ-HSA-TAP had significantly increased systolic volume compared to naïve hearts (Figure 5G; 54.8 ± 9.1 PBS; 55.6 ± 9.5 non-targ-HSA-TAP *vs.* 25.4 ± 5.7 naïve; P = 0.0006 and P = 0.0004 respectively). No difference in systolic volume was observed between naïve mice and targ-HSA-TAP mice (Figure 5G; 25.4 ± 5.7 *vs.* 29.6 ± 4.1; P = ns). Similar results were obtained for diastolic volume (Figure 5H), indicating that targ-HSA-TAP prevented left ventricle enlargement.

**Figure 6:**
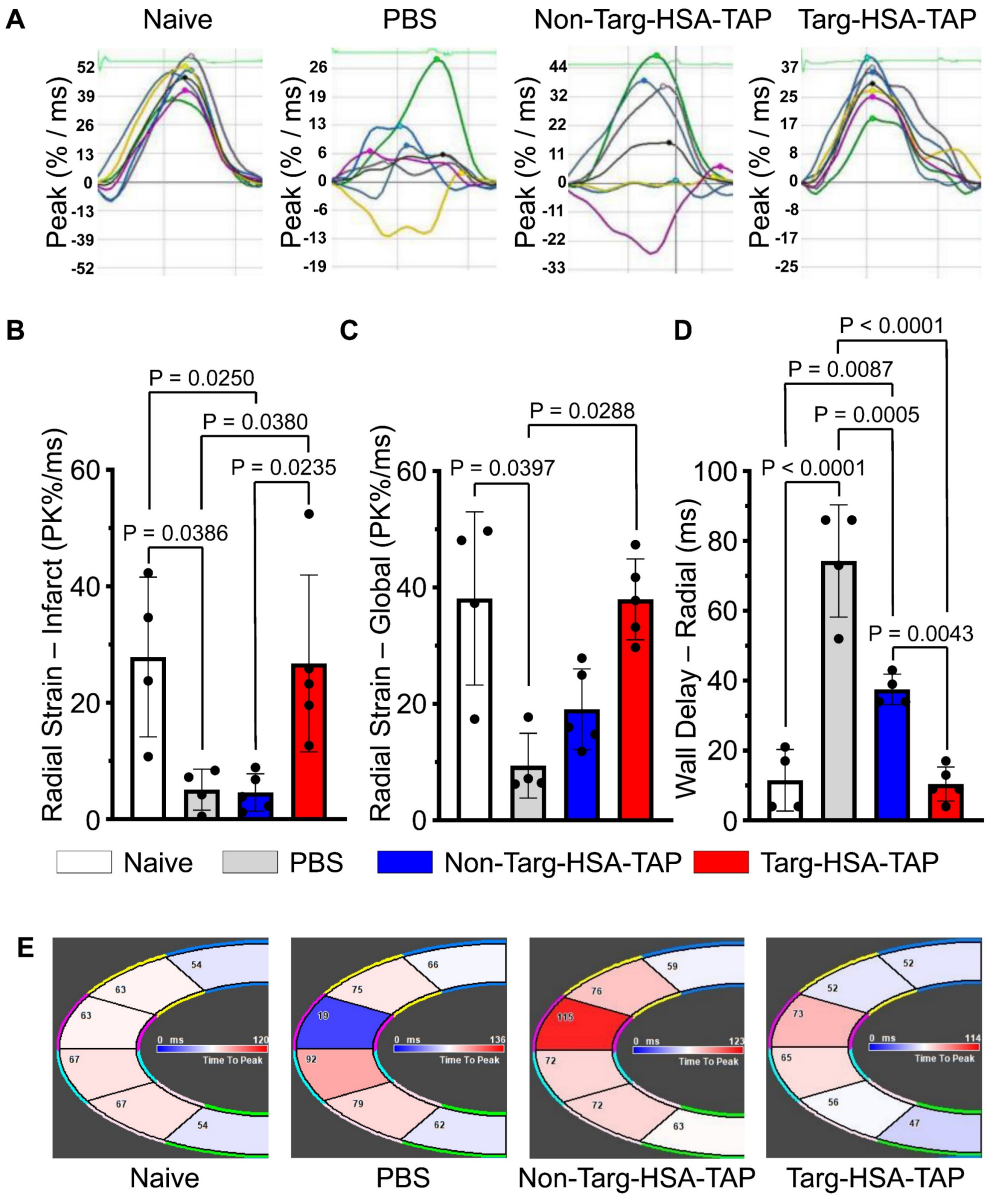
** Heart pumped synchronously after targ-HSA-TAP treatment in a mouse model of ischemia/reperfusion (I/R). A.** Representative images of radial strain curves obtained from VevoStrain analysis software illustrating strain measurements over time. Six heart regions are represented by six colored lines. Average (global) strain at each time point is shown as a black line in each graph. **B.** Bar chart shows PBS and non-targ-HSA-TAP groups had significantly decrease radial strain in the infarct area (anterior apex) as compared to targ-HSA-TAP and naïve groups, n = 4-5. **C.** Bar chart shows radial strain globally, where targ-HSA-TAP group demonstrated preservation and maintained healthy muscles comparable to naïve group, n = 4-5. **D.** Maximum opposite-wall delay and **E.** Representative images show significant increases in time delay for PBS-treated mice as compared to targ-HSA-TAP treated mice, n = 4-5. Numerical results shown as mean ± SD. Data in B and D analyzed using one-way ANOVA with Tukey's post-test analysis. Data in C analyzed using Kruskal-Wallis test with Dunn's multiple comparisons.

Strain analysis is a well-established clinical method and a more sensitive way to determine cardiac function 6. The heart is divided into six sections to assess myocardial contractility and their lines relate to synchronicity (Figure S5); hence when the lines are disorganized, this equates to irregularity in muscle movements (Figure 6A). Four weeks post-I/R, pathological changes in infarct (anterior apex) and global areas of radial strain were observed in the PBS and non-targ-HSA-TAP treated groups as compared to the targ-HSA-TAP group (Figure 6B-C). Compared to the naïve hearts, radial strain in the infarct areas was reduced in the PBS and non-targ-HSA-TAP hearts (Figure 6B; 27.9 ± 13.7 naïve *vs.* 5.1 ± 3.5 PBS, 4.6 ± 3.2 non-targ-HSA-TAP; P = 0.0386 and P = 0.0250 respectively), but preserved in the targ-HSA-TAP group (Figure 6B; 27.9 ± 13.7 *vs.* 26.7 ± 15.2; P = ns). Similar results were observed for radial strain in global areas (Figure 6C). A significant decrease in wall delay was observed in the targ-HSA-TAP treated group as compared to the PBS and non-targ-HSA-TAP groups, suggesting the left ventricle of the targ-HSA-TAP treated hearts pumped more synchronously at 4 weeks following I/R (Figure 6D and 6E; 10.4 ± 4.9 targ-HSA-TAP *vs.* 74.3 ± 16.1 PBS, 37.5 ± 4.4 non-targ-HSA-TAP; P < 0.0001 and P = 0.0043 respectively).

**Figure 7:**
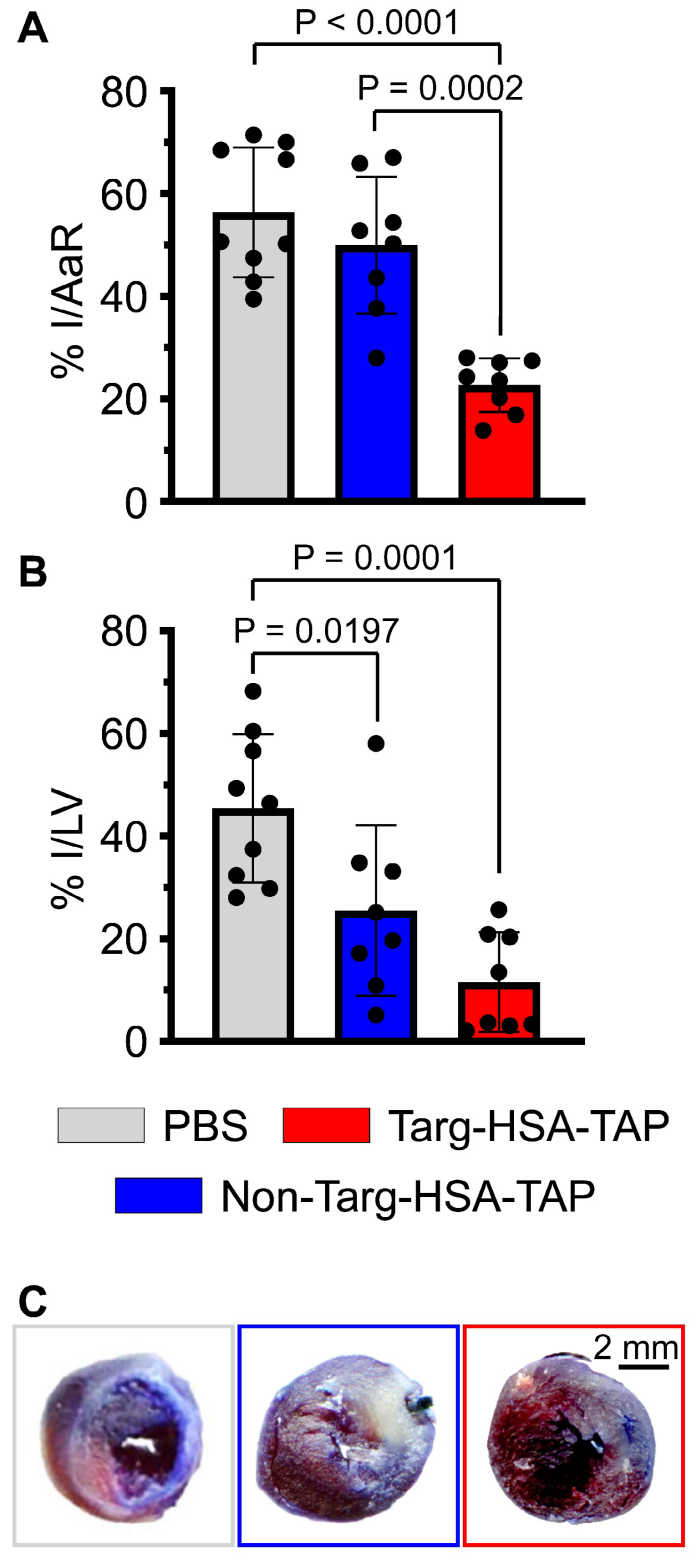
** Targ-HSA-TAP treatment ameliorated cardiac remodeling in a mouse model of ischemia/reperfusion (I/R). A.** Targ-HSA-TAP decreased infarct size (I)/area at risk (AaR) in hearts as compared to PBS control and non-targ-HSA-TAP, n = 8-9. **B.** A significant reduction of infarct in left ventricle (LV) was measured for targ-HSA-TAP treated group as compared to PBS control group, n = 8-9. **C.** Representative images of Evans blue/TTC (triphenyltetrazolium chloride) staining of heart slices 4 weeks post-I/R. Numerical results shown as mean ± SD. Assays analyzed using one-way ANOVA with Tukey's post-test analysis.

Histological data analyzed at 4 weeks post cardiac I/R demonstrated a significant decrease in the size of the infarct (I)/area at risk (AaR) in the targ-HSA-TAP group as compared to the PBS and non-targ-HSA-TAP groups (Figure 7A; 22.7 ± 5.2 targ-HSA-TAP *vs.* 56.3 ± 12.7 PBS, 49.9 ± 13.3 non-targ-HSA-TAP; P < 0.0001 and P = 0.0002 respectively). Treatment with targ-HSA-TAP also resulted in a reduction in the infarct size (Figure 7B and 7C; 5.8 ± 4.8 *vs.* 22.7 ± 7.2 PBS; P = 0.0001). Thus, treatment with targ-HSA-TAP 10 h prior to I/R significantly reduced infarct size and strongly correlated with the preserved cardiac function on ultrasound imaging.

## Discussion

In this study, we have generated and characterized a novel antithrombotic combination drug with extended half-life and potential thromboprophylactic applications. Targ-HSA-TAP combines activated-platelet-targeting and FXa inhibition at the site of clot formation. When administered via s.c. injection up to 16 h before vascular injury, targ-HSA-TAP can reduce acute thrombus formation without impacting hemostasis, highlighting its potential for the secondary prevention of cardiovascular events. Importantly, in addition to its potent antithrombotic effects, targ-HSA-TAP preserves cardiac function and reduces infarct size after reperfusion, demonstrating its potential to provide benefits for recovery in the case of an MI. Overall, we demonstrate that this novel drug: (1) can be administered at low systemic concentrations, thereby preventing unwanted bleeding side effects; (2) targets the site of thrombus formation to enhance localized drug potency; and (3) has an increased circulation time, preventing the necessity for repeated administration of drugs and providing the potential for platelet-targeted thromboprophylactic strategies.

Thrombosis is a major factor contributing to disability and mortality worldwide. One notable drawback of clinically approved pharmacological therapies, including a combination of antiplatelet and/or anticoagulant drugs, is their strong association with bleeding complications. In fact, iatrogenic bleeding complications cause significant morbidity and mortality 16-18. In our previous studies, we have pioneered the development of multiple dual-action platelet-targeted drugs and have demonstrated their efficacy in thrombolysis and anticoagulation across various disease models 6,10-12. In particular, the parent drug of our novel construct, targ-TAP, has demonstrated its potential for broad application in arterial and venous thrombosis without compromising hemostasis. However, the molecular size of targ-TAP is 37 kDa, resulting in a relatively short duration of action of around 4 h via s.c. administration 10. The limited circulation time of targ-TAP is a significant drawback because it requires frequent administration to provide therapeutic benefit; therefore, this drug is not ideal for long-term secondary prevention of recurrent thrombotic events.

To generate a drug with a long half-life suitable for thromboprophylactic applications, we increased the size of our scFv drug construct and added further half-life-extending properties. As an approach successfully used in the pharmaceutical industry, we chose to use recombinant fusion technology to genetically incorporate HSA between the scFv and the TAP. Other approaches for circulating half-life extensions have potential disadvantages and were not pursued. Chemical conjugation, achieved by targeting and modifying specific amino acids, comes with the risk of altering the structure and/or characteristics of the binding pocket of the scFv_Targ_, potentially affecting its binding affinity and specificity 19,20. Covalent attachment of drugs to scFv can also introduce stability issues, including susceptibility to degradation or cleavage at the conjugation site, which may affect the overall stability and half-life 20. Modification of amino acids may also reduce the therapeutic activity of TAP. Chemical conjugation methods can also lead to heterogeneity, where the different conjugation sites and stoichiometries of drug attachment may generate a mixture of scFv drug constructs with reduced and varying drug efficacies 19,20. This heterogeneity can also complicate the characterization of the scFv drug constructs, impacting the overall consistency and therapeutic predictability, making dosing and efficacy studies challenging 20. In contrast, our genetic modification strategy resulted in a consistent scFv-HSA drug fusion construct with one targeting moiety, one HSA to increase half-life circulation, and one TAP drug.

Furthermore, the intrinsic ubiquity of HSA in plasma, coupled with its extended circulating half-life, positions it as an attractive candidate for improving drug pharmacokinetics and indeed it has been used in a multitude of medical and pharmaceutical applications 14. HSA can serve as a versatile scaffold or carrier for drugs and therapeutic agents, extending their presence in circulation. The intrinsic nature of HSA as a naturally occurring protein imparts an additional layer of utility, including shielding drugs from degradation, bolstering their stability, and curtailing the risk of drug resistance, all of which are particularly evident in oncological studies 21,22. Furthermore, HSA possesses immunosuppressive properties through reducing complement cascade activation, minimizing the risk of immunogenicity 23,24. Beyond this, HSA has found application in the coating of blood-contacting medical devices, and HSA-associated drug fusion proteins have demonstrated efficacy in preventing platelet activation 25.

The genetic fusion of HSA with therapeutic agents has resulted in half-life extensions, such as with Kunitz protease inhibitor (KPI), factor VIIa (FVIIa), and bispecific antibodies targeting epidermal growth factor receptors (ERBBErbB2 and ErbB3) 26-30. KPI, with its small molecular weight of 6 kDa, benefits substantially from the addition of HSA, allowing the HSA-KPI fusion protein to exhibit effectiveness in reducing venous and arterial thrombosis when administered 1 h prior to disease induction 26. Weimer *et al.* demonstrated that the half-life of HSA-FVIIa increased from a modest 2.5 h (without HSA) to 15-17.5 h in rat models 27. Studies have also shown that a 55 kDa bispecific antibody targeting ErbB2 and ErbB3 provided anti-tumour activity *in vitro* and *in vivo* but had a half-life of 4 to 5 h, posing challenges in attaining the requisite serum levels for therapeutic activity in oncology patients 28-30. McDonagh *et al.* demonstrated that the HSA-fused ErbB2/ErbB3-bispecific antibody protein (MM-111) had a half-life ranging from 16 to 20 h in murine models 29. These *in vivo* data corroborate our findings on targ-HSA-TAP as a thromboprophylactic agent in our thrombosis model, underscoring the reliability and consistency of HSA-conjugated drugs. Furthermore, subsequent research affirmed that the half-life of MM-111 in cynomolgus monkeys extended to approximately 99 h (approximately 4.1 days) 29. However, given that complete cross-species pharmacokinetic congruence is not achieved, a further extended half-life is expected in humans.

An increased molecular weight after incorporation of HSA is not the only reason for an extended half-life of drugs. In the human context, HSA engages in diverse cellular interactions, including interactions with GP60, GP30, GP18, the megalin/cubilin complex, and, most importantly, FcRn receptors 13,15,31-34. It is crucial to note that the prolonged half-life of HSA is substantially associated with FcRn receptor-mediated HSA recycling 15. However, in murine models HSA is not as adeptly recognized by FcRn receptors, thereby rendering the extended circulating time of fusion drugs, including our targ-HSA-TAP, primarily dependent on the augmented molecular weight and, thus, the pharmacological advantage of an HSA-coupled drug expected in patients is not visible in mice. In mice the half-life of HSA is approximately 21 h, a notable contrast to the human half-life of 19 days 35. As a result, the 16 h thromboprophylactic effect observed in our murine studies is anticipated to reflect a much longer half-life in the human context. Indeed, clinical trials have provided strong evidence for the prolonged pharmacokinetics of HSA fusion proteins. An exemplary case is the fusion of factor IX (FIX) with HSA (rIX-FP), in which the terminal half-life of rIX-FP varied between 14.8 and 36.2 h across animal species, including mice, rats, and rabbits; however, in human patients a half-life of approximately 102 h was demonstrated 36,37. In a more recent clinical trial, Mancuso *et al.* elucidated the mean terminal half-life of rIX-FP (100 IU/kg) to be 143.2 h and, more importantly, it exhibited a prophylactic effect with an extended dosing interval of up to 21 days in patients with hemophilia B 38. Furthermore, albiglutide, an FDA-approved therapeutic for type 2 diabetes, comprises two glucagon-like peptide-1 (GLP-1) molecules fused with HSA. Initially, the *in vivo* half-life of GLP-1 is a mere 1-2 min, depending on the species, due to the dipeptidyl peptidase-4-induced enzymatic degradation and subsequent renal elimination 39-41. Conjugation with HSA extends the half-life to approximately 6-8 days in humans, rendering it suitable for a once-weekly administration regimen 42,43. Our targ-HSA-TAP approach demonstrates significant promise for clinical translation, supported by the established clinical use of several HSA drugs 38,42,44-46. Following an MI, patients are at high risk of secondary thrombotic events, highlighting the need for ongoing antithrombotic treatment. Targ-HSA-TAP, for example, holds significant promise to be used in secondary antithrombotic prophylaxis for patients at a high risk of reinfarction.

In our *in vivo* experiments, targ-HSA-TAP and targ-TAP were administered at equimolar ratios to account for the molecular weight of HSA and to ensure equivalent doses of antiplatelet/anticoagulant activity. In mice experiencing an acute thrombotic injury in the carotid artery, we observed a prolonged antithrombotic effect for up to 16 h after s.c. injection of targ-HSA-TAP. In contrast, the antithrombotic benefit of targ-TAP was only maintained for up to 4 h. These data demonstrate that the incorporation of HSA successfully extended the duration of antithrombotic effects for employment as a long-acting thromboprophylactic approach. This highlights the potential for targ-HSA-TAP to be used to prevent thrombosis in high-risk patients, thereby preventing events such as MI and stroke from occurring.

Despite anticoagulation and antiplatelet therapy, recurrent cardiovascular events are common, occurring in up to 18.5% of patients 4. Since we have previously shown that platelets are involved in cardiac I/R injury and can be targeted via our single-chain antibody against activated GPIIb/IIIa 47-49, we hypothesized that targ-HSA-TAP could also prevent cardiac I/R injury. We used a mouse model of cardiac I/R injury (ligation of the LAD for 1 h followed by reperfusion) which mimics patients undergoing percutaneous coronary intervention (PCI) as a treatment for MI. Previous attempts to use targ-TAP as a therapy via i.v. administration at reperfusion demonstrated its ability to reduce infarct area 12. In this study, our data show that s.c. injection of targ-HSA-TAP 10 h before cardiac I/R injury preserved cardiac function, reduced cardiac deformation, and decreased infarct size. This result reflects the novel and paradigm-changing prophylactic approach of targ-HSA-TAP as a second layer of protection for myocardial I/R injury and the ability of this drug to reduce cardiac damage in the event of MI.

Beyond the scope of MI prevention and treatment, HSA-conjugated activated-platelet-targeted drug therapy can be applied to various other thrombotic diseases. For instance, patients with atrial fibrillation presenting with acute coronary syndrome or undergoing PCI with stent placement are prescribed a so-called triple therapy (TT) comprising an oral anticoagulant and dual antiplatelet treatment 50. Notably, TT is associated with significantly elevated bleeding risk, which intensifies with prolonged use of up to 12% 51,52. Targ-HSA-TAP has the potential to offer an equivalent level of protection for an extended duration of action while retaining normal hemostasis. Also, the prophylaxis of venous thromboembolism (VTE), particularly in patients at a high risk of bleeding, remains a high clinical priority based on the associated morbidity and mortality 53. Given the promising thromboprophylatic effect of targ-TAP in reducing VTE in murine models without bleeding issues 10, targ-HSA-TAP is also attractive for long-term secondary VTE prophylaxis.

In conclusion, we have demonstrated that targ-HSA-TAP provides effective thromboprophylaxis for up to 16 h when administrated s.c. in mice. Based on the longer half-life expected in humans, targ-HSA-TAP holds particular promise as a thromboprophylaxis agent in high-risk patients and its downstream clinical sequelae, MI and stroke, without associated bleeding risk. Additionally, in the event that MI occurs, targ-HSA-TAP given as a long-acting thromboprophylactic drug represents a second line of defense to preserve cardiac function and reduce infarct size. Its targeting ability enables a low dose to be administered and the drug will home in on the site of thrombosis, thereby increasing its potency at the area of need. The dose required for effective thromboprophylaxis is not associated with an increase in bleeding risk, in contrast to all currently clinically used antithrombotic drugs, thereby providing effective and safe dual antiplatelet and anticoagulation therapy.

## Supplementary Material

Supplementary figures and video legends.

Supplementary video 1.

Supplementary video 2.

Supplementary video 3.

Supplementary video 4.
