# Supplementary material for: Platelet-targeted thromboprophylaxis with a human serum albumin fusion drug: Preventing thrombosis and reducing cardiac ischemia/reperfusion injurywithout bleeding complications
Source: Theranostics. 2024 May 19;14(8):3267–81. doi: 10.7150/thno.97517 (PMC11155409; doi:10.7150/thno.97517)
Supplement: Supplementary file 1 — Supplementary figures and video legends. [file thnov14p3267s1.pdf]

Supplementary data

Platelet-targeted thromboprophylaxis with a human serum albumin fusion drug: Preventing thrombosis and reducing cardiac ischemia/reperfusion injury without bleeding complications

Yuyang Song[1,2,*], Laura A. Bienvenu[1,2,3,*], Viktoria Bongcaron[1,2,4], Shania A. Prijaya[1,2,4], Ana C. Maluenda[4], Aidan P. G. Walsh[1], James D. McFayden[2,4], Geoffrey A. Pietersz[2,4], Karlheinz Peter[2,4,5,#,^], Xiaowei Wang[1,2,3,5,#,^]

[1] Molecular Imaging and Theranostics Laboratory, Baker Heart and Diabetes Institute, Melbourne, VIC, Australia.

[2] Department of Cardiometabolic Health, University of Melbourne, VIC, Australia.

[3] Baker Department of Cardiovascular Research, Translational and Implementation, La Trobe University, Melbourne, Australia.

[4] Atherothrombosis and Vascular Biology Laboratory, Baker Heart and Diabetes Institute, Melbourne, VIC, Australia.

[5] Department of Medicine, Monash University, Melbourne, VIC, Australia.

[*]equally contributing first authors

[#]equally contributing senior authors

[^]Corresponding authors

Supplementary figures

Figure S1

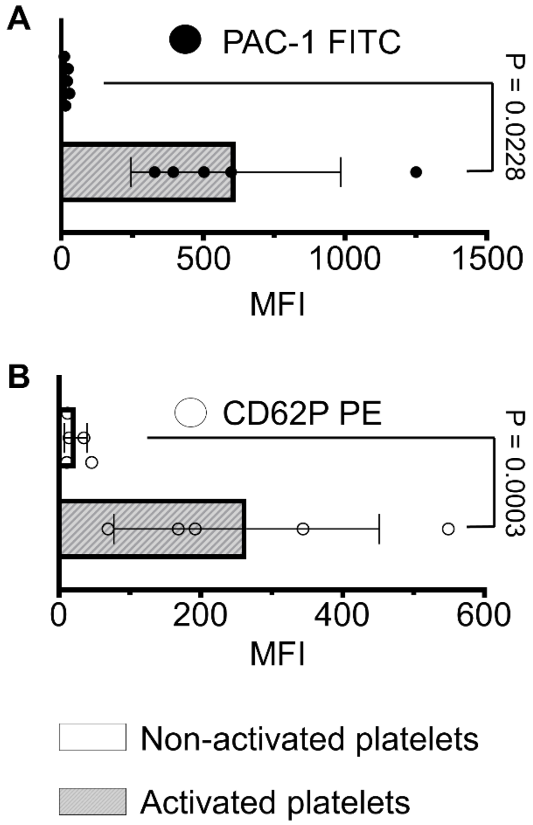

Figure S1. Flow cytometry assay demonstrating platelet-activation markers bound to ADP-activated platelets Bar charts display mean fluorescence intensity (MFI) values of 5 independent experiments. **A.** PAC-1 FITC; and **B.** CD62P PE bound to activated platelets and increased MFI, but not with non-activated platelets. Data analyzed using Welch's t-test.

Figure S2

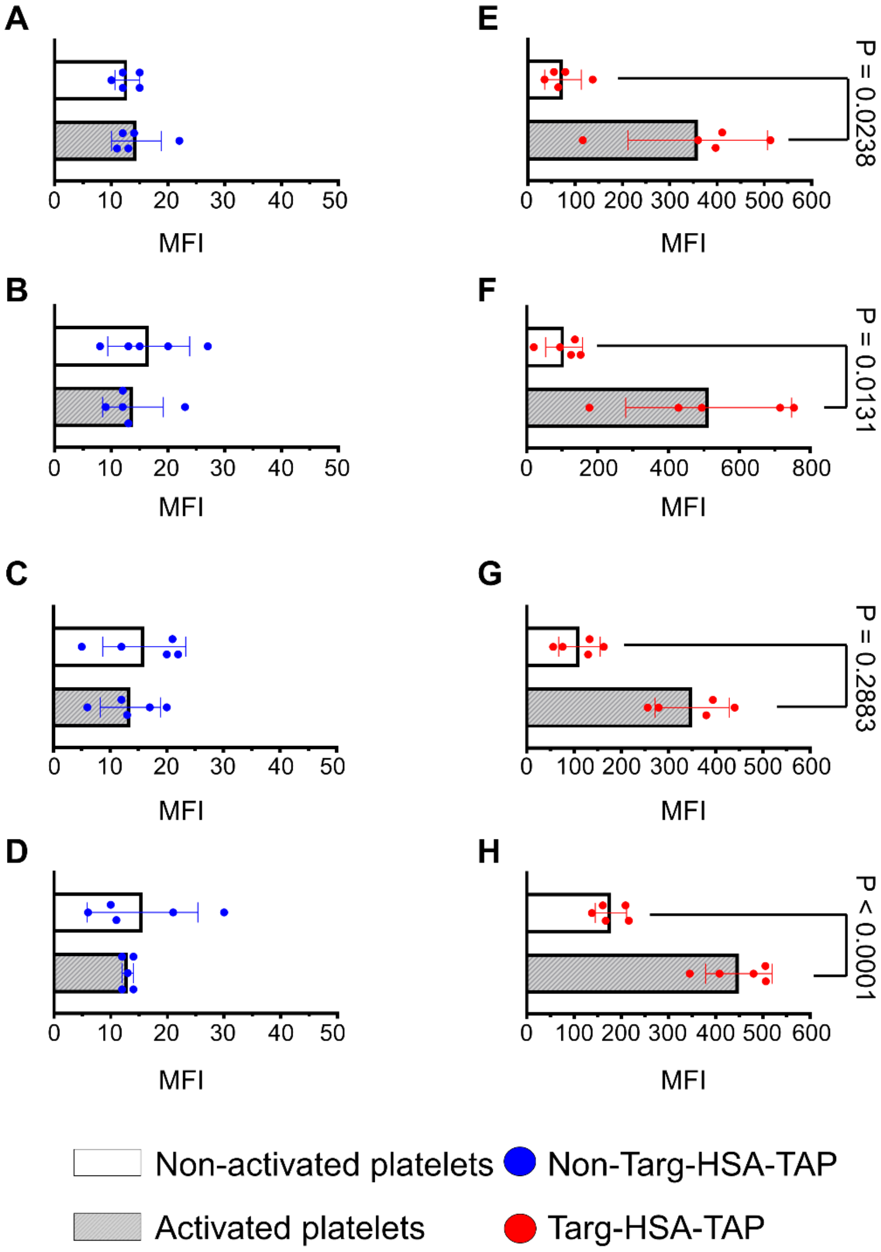

Figure S2. Targ-HSA-TAP bound specifically to activated human platelets. Flow cytometry assay was conducted using anti-Penta-His AlexaFluor 488 antibody, which bound our constructs. Platelets were either non-activated or activated with ADP. Bar charts display mean fluorescence intensity (MFI) values of 5 independent experiments. No binding was observed in samples with non-targ-HSA-TAP at **A.** 0.5 µg/ml; **B.** 1 µg/ml; **C.** 2 µg/ml; and **D.** 5 µg/ml. Specific binding to activated platelets was observed in samples with targ-HSA-TAP

at **E.** 0.5 μg/ml; **F.** 1 μg/ml; **G.** 2 μg/ml; and **H.** 5 μg/ml. Data in A–C, G, and H analyzed using Student's t-test. Data in D–F analyzed using Welch's t-test.

Figure S3

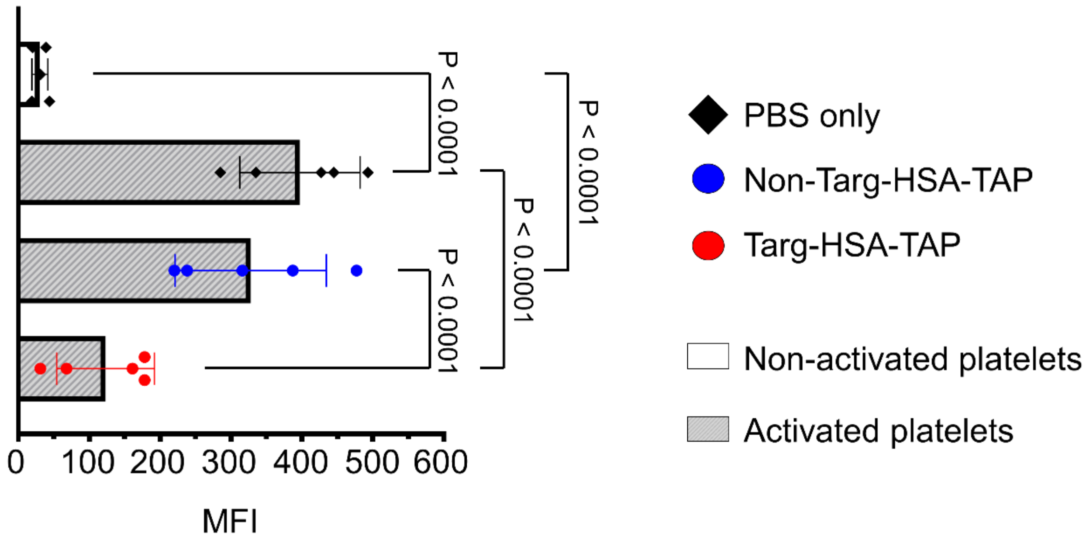

Figure S3. Targ-HSA-TAP and PAC-1 targeted activated glycoprotein IIb/IIIa receptors competitively. Bar charts show mean fluorescence intensity (MFI) values of 5 independent experiments. PAC1-FITC bound to activated platelets when incubated with PBS (without constructs) or non-targ-HSA-TAP (2 μg/mL). After incubating activated platelets with a small dose of targ-HSA-TAP (2 μg/mL), most binding sites were blocked, resulting in less binding with PAC1-FITC. Finding is concentration-dependent. Data analyzed using one-way ANOVA with Tukey’s post-test analysis.

Figure S4

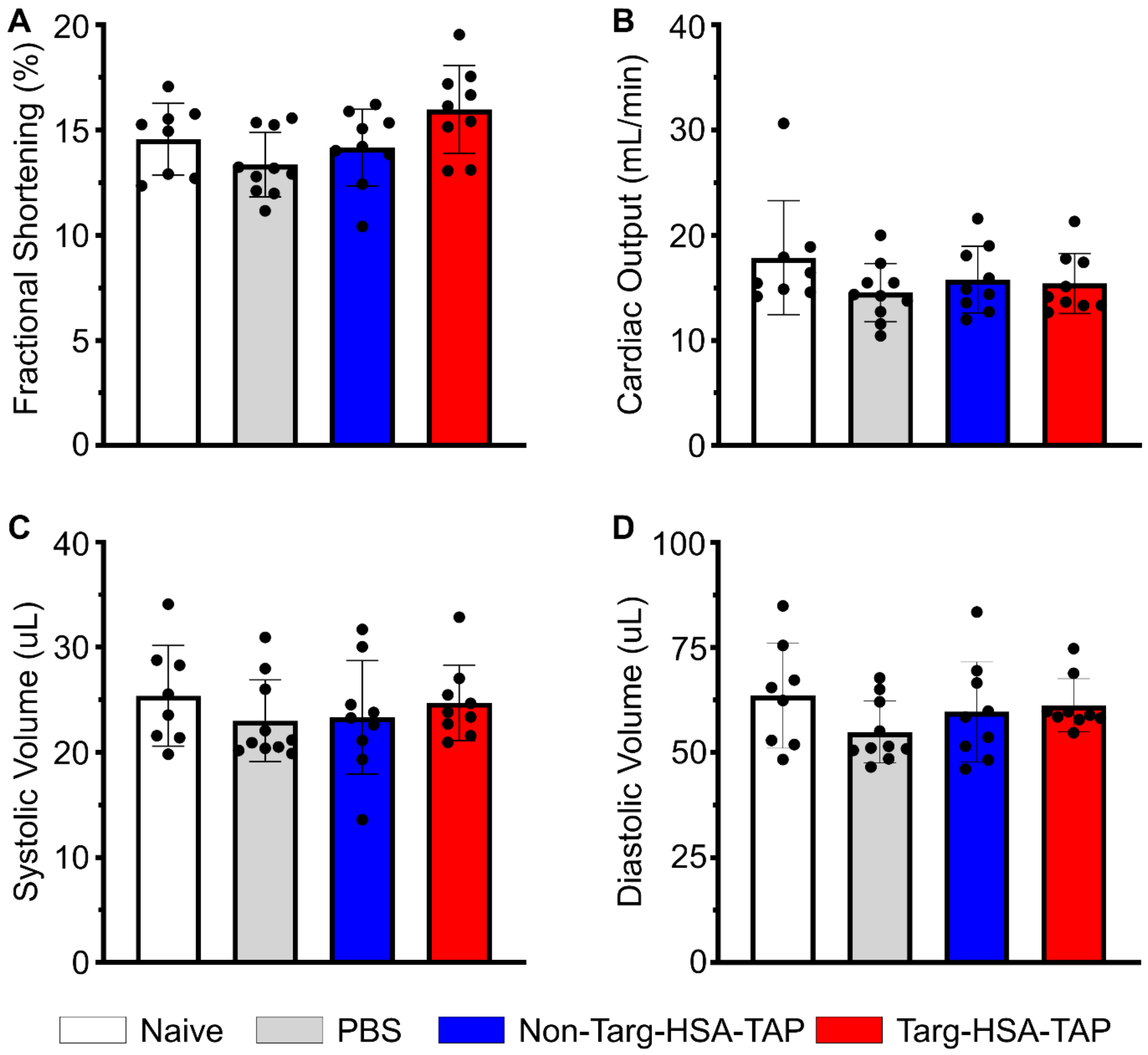

Figure S4. Baseline echocardiography data were similar across all groups. Cardiac parameters were measured from parasternal long-axis B-mode images. **A.** Fractional shortening; **B.** Cardiac output (CO); **C.** Systolic volume; and **D.** Diastolic volume, all n = 8–10. Numerical results shown as mean ± SD. Data in A–C analyzed using one-way ANOVA with Tukey's post-test analysis. Data in D analyzed using Brown–Forsythe's ANOVA test with Dunnett's T3 multiple comparisons.

Figure S5

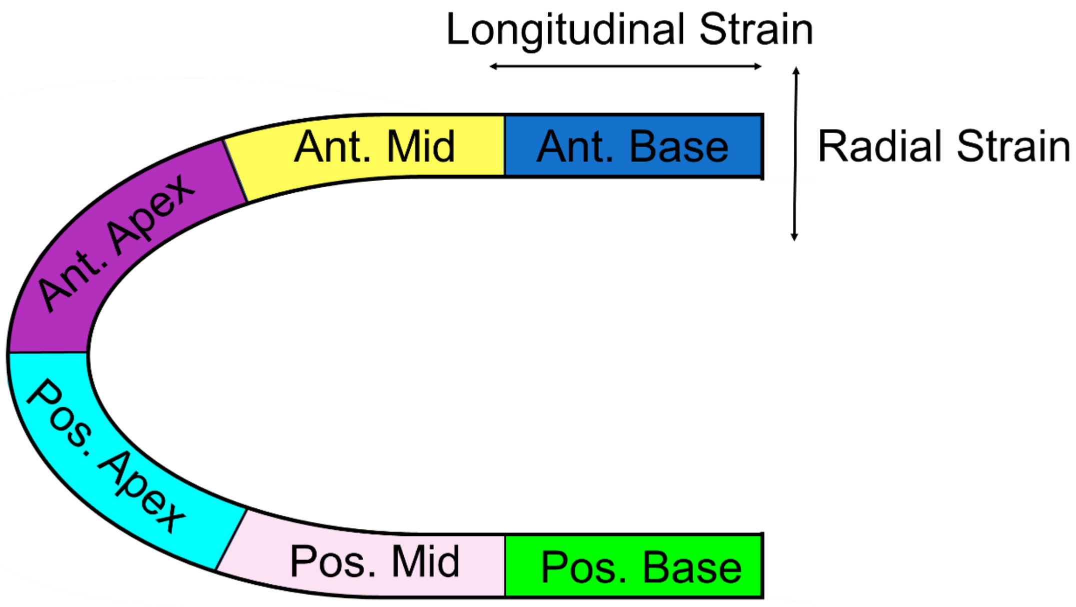

Figure S5. Systemic diagram showing left ventricle of mouse heart in parasternal long-axis view. To obtain strain analysis data, left ventricle is divided into six sections and colored lines correlate to each section. Strain analysis calculated by tracking six anatomical locations: Anterior (Ant.) Base, Ant. Mid, Ant. Apex, Posterior (Pos.) Apex, Pos. Mid, and Pos. Base.

Video legends

Video S1: *In vivo* echocardiographic examination of a naïve mouse. The video was imaged from a parasternal long-axis view via B-mode and shows normal wall movement and cardiac contractility at Week 4.

Video S2: *In vivo* echocardiographic examination of a PBS-treated mouse. The video was imaged from a parasternal long-axis view via B-mode and shows impaired wall movement and reduced cardiac contractility at Week 4 post-MI.

Video S3: *In vivo* echocardiographic examination of a non-targ-HSA-TAP treated mouse. The video was imaged from a parasternal long-axis view via B-mode and shows impaired wall movement and reduced cardiac contractility at Week 4 post-MI.

Video S4: *In vivo* echocardiographic examination of a targ-HSA-TAP treated mouse. The video was imaged from a parasternal long-axis view via B-mode and shows normal wall movement and preserved cardiac contractility at Week 4 post-MI.
